# “Let’s Chat!” Improving Emergency Department Staff Satisfaction with the Medication Reconciliation Process

**DOI:** 10.5811/westjem.18324

**Published:** 2024-05-21

**Authors:** Kurt Schwieters, Richard Voigt, Suzette McDonald, Lori Scanlan-Hanson, Breanna Norman, Erin Larson, Alexis Garcia, Bo Madsen, Maria Rudis, Fernanda Bellolio, Sara Hevesi

**Affiliations:** * Mayo Clinic, Department of Emergency Medicine, Rochester, Minnesota; † Idaho College of Osteopathic Medicine, Meridian, Idaho; ‡ Mayo Clinic, Department of Nursing, Rochester, Minnesota; § Mayo Clinic, Department of Pharmacy, Rochester, Minnesota

## Abstract

**Introduction:**

Patients who stay in the emergency department (ED) for prolonged periods of time require verification of home medications, a process known as medication reconciliation. The complex nature of medication reconciliation can lead to adverse events and staff dissatisfaction. A multidisciplinary team was formed to improve accuracy, timing, and staff satisfaction with the medication reconciliation process.

**Methods:**

Between November 2021–January 2022, stakeholders were surveyed to identify gaps in the medication reconciliation process. This project implemented education on role-specific tasks, as well as a “Let’s chat!” huddle, bringing together the entire care team to perform medication reconciliation. We used real-time evaluations by frontline staff to evaluate effectiveness during plan- do-study-act cycles and obtain feedback. Following the implementation period, stakeholders completed the post-intervention survey between June-July 2022, using a 4-point Likert scale (0 = very dissatisfied to 3 = very satisfied). We calculated the change in staff satisfaction from pre-intervention to post-intervention. Differences in proportions and 95% confidence intervals are reported. This study adhered to the Standards for Quality Improvement Reporting Excellence (SQUIRE 2.0) and followed the Lean Six Sigma rapid cycle process improvement (define-measure-analyze-improve-control).

**Results:**

A total of 111 front-line ED staff (physicians, nurse practitioners, physician assistants, pharmacists, nurses) completed the pre-intervention survey (of 350 ED staff, corresponding to a 31.7% response rate), and 89 stakeholders completed the post-intervention survey (a 25.4% response rate). Subjective feedback from staff identifying causes of low satisfaction with the initial process included the following: complexity of process; unclear delineation of staff roles; time burden to completion; high patient volume; and lack of standardized communication of task completion. Overall satisfaction improved after the intervention. The greatest improvement was seen in the correct medication (difference 20.7%, confidence interval [CI] 6.3–33.9%, *P* < 0.01), correct dose (25.6%, CI 11.4–38.6%, *P* < 0.001) and time last taken (24.5%, CI 11.4–37.0%, *P* < 0.001).

**Conclusion:**

There is a steep learning curve to educate multidisciplinary staff on a new process and implement the associated changes. With goals to impact the safety of our patients and reduce negative outcomes, engagement and awareness of the team involved in the medication reconciliation process is critical to improve staff satisfaction.

Population Health Research Capsule What do we already know about this issue?

*Medication reconciliation for boarding ED patients is complex and can lead to adverse events and staff dissatisfaction.*

What was the research question?

*How can we improve the process of medication reconciliation for boarding patients?*

What was the major finding of the study?

*After implementation of the medication reconciliation improvement project, staff satisfaction score improved an average of 20–25.6% for correct medication, dose, and time last taken.*

How does this improve population health?

*Having a streamlined process for medication reconciliation and ordering ensures that all patients accurately receive their home medications while boarding in the ED.*

## INTRODUCTION

### Problem Description

There is a shortage of inpatient beds in our nation’s hospitals. This shortage results in the frequent practice of retaining to-be-admitted patients in the ED until their inpatient bed becomes available. This practice is known as “ED boarding.”^1^ Patients subjected to ED boarding sustain a prolonged ED length of stay (LOS). In many instances, the ED LOS becomes so lengthy that these patients’ usual, or “home,” medications must be correctly administered while they remain in the ED,^2^ rather than being administered only after the patient arrives to their inpatient bed. To enable accurate administration of these “home” medications, the process of “medication reconciliation” must occur within the ED.

“Medication reconciliation” is the process of verification of the names of the patient’s usual medications, as well as their dosages and times of administration. Medication reconciliation for “boarded” patient at our institution has become the responsibility of the ED staff, who also must correctly obtain and administer medications newly ordered by the emergency physician. The ED medication history and reconciliation process is complex and error prone,^3^ particularly in the setting of competing, urgent priorities in the ED, and results in a high risk of adverse patient outcomes.^4^ We identified a staff satisfaction gap in the process of medication reconciliation in our ED and sought to improve this process.

### Available Knowledge

All patients admitted to the hospital require a medication reconciliation, defined by the Joint Commission as the process of reviewing and confirming medications that a patient is currently taking to the medications that are ordered for the patient.^5^^,^^6^ To avoid errors, the Joint Commission National Patient Safety Goal requires that a good faith effort be made to obtain complete medication information from the patient. Despite this effort, errors still occur.^7^ A medication discrepancy, defined as inconsistencies between two or more medication lists, impacts nearly all patients admitted to the hospital, increasing potential harm to patients.^8^ Adverse drug events (ADE) due to unintentional discrepancies in the admission medication list have been cited as the most common cause of preventable drug events.^9^ If not recognized early, medication discrepancies can lead to an increased risk of readmissions, ED visits, and prolonged hospital stays.^9^

Allocating a member of the pharmacist team to handle this specific task, as is done with patients admitted to inpatient beds, could ensure safe and timely medication reconciliation, subsequently improving patient care.^10^ In the state of Minnesota, however, the law precludes pharmacy technicians from obtaining medication histories and taking responsibility for medication reconciliation.^11^ Using pharmacists to obtain medication histories and perform medication reconciliation is an option in some EDs but not in ours. This limitation is not unique to our facility, because in Minnesota pharmacy technicians are not allowed to obtain or review a patient’s medication list. Further, given that there is a national pharmacist shortage^12^ and that practice advisories arising from the American College of Emergency Physicians (ACEP) and other organizations have long stated that it is preferable to have pharmacists focus their clinical efforts on bedside patient care,^13^ we determined that non-pharmacist emergency clinicians must become involved in the process of medication reconciliation at our facility.

### Rationale

At our institution, there is low staff satisfaction with the current medication history, reconciliation and home medication ordering process for patients with extended LOS in our ED observation unit (EDOU) and behavioral health (BH) area. Standard processes for performing medication histories and ordering home medications as used in the inpatient setting are difficult in the ED given other priorities and urgent tasks in this environment, the time required, multiple interruptions, and the lack of a dedicated role to perform the task.^14^ Dissatisfaction with the process may contribute to delays, inaccuracies, and safety events. Interprofessional training modules for taking medication histories and medication reconciliation in the ED have been shown to improve employee communication, behavior, knowledge, and attitude.^15^ Despite previous educational initiatives, safety events related to medication histories reconciliation persist. Thus, we sought to newly assess our current ED staff satisfaction to further improve the process for EDOU and BH patients.^15^

### Specific Aims

In this project we aimed to assess and improve ED staff satisfaction with the medication reconciliation process for patients with prolonged ED stay, including EDOU and BH boarding patients, by 20%.

## METHODS

This quality improvement (QI) initiative was a before-and-after study and considered to be exempt from institutional review board review. We followed the Standards for Quality Improvement Reporting Excellence: (SQUIRE 2.0) standardized methodological guidelines. We used the Lean Six Sigma rapid cycle process improvement to overcome barriers to protocol use and fidelity with the define-measure-analyze-improve- control) framework.^16^ In this study we used voluntarily provided, anonymous staff survey information. Our pre-intervention survey was sent out in November 2021, and our post-intervention survey was completed in July 2022.

### Context

Stakeholders included ED front-line staff (ie, attending physicians, emergency medicine [EM] residents, nurse practitioners [NP], physician assistants [PA], pharmacists, registered nurses [RN], care team assistants [CTA], ED psychiatry consult team [psychiatry-specific physician, resident, and NP or PA]), ED quality staff, and patients and their families. The CTAs are ED employees who facilitate moving patients on the electronic health record (EHR) track board, communicating with consulting services, scheduling outpatient appointments, and in general having overall awareness of patient flow throughout the department. Our study team included representative members of the various stakeholder groups, all of whom volunteered their time to this project.

Our institution is an academic medical center embedded within a larger healthcare system in the Midwest. We have a volume of 78,000 visits per year and are a Level I trauma and stroke center. Of the 70 beds in the ED, four are dedicated for BH patients and nine are used for ED observation. We have a three-year EM residency training program with nine residents per year as well as an NP/PA EM fellowship. Various resident programs rotate through the ED. We have 12 ED-specific NPs or PAs. Our pharmacists provide 24/7 coverage to our department, and we have a pharmacy residency program with one fellow per year.

The medication history and reconciliation process used in our ED at the time this study was initiated lacked a clear delineation of each clinician’s role in the process. A need existed for each patient’s medication list to be verified, but our procedures did not define which ED frontline staff must perform this task. All patients who will be admitted and are EDOU or BH boarding require a medication reconciliation.

### Interventions

**Figure 1.**
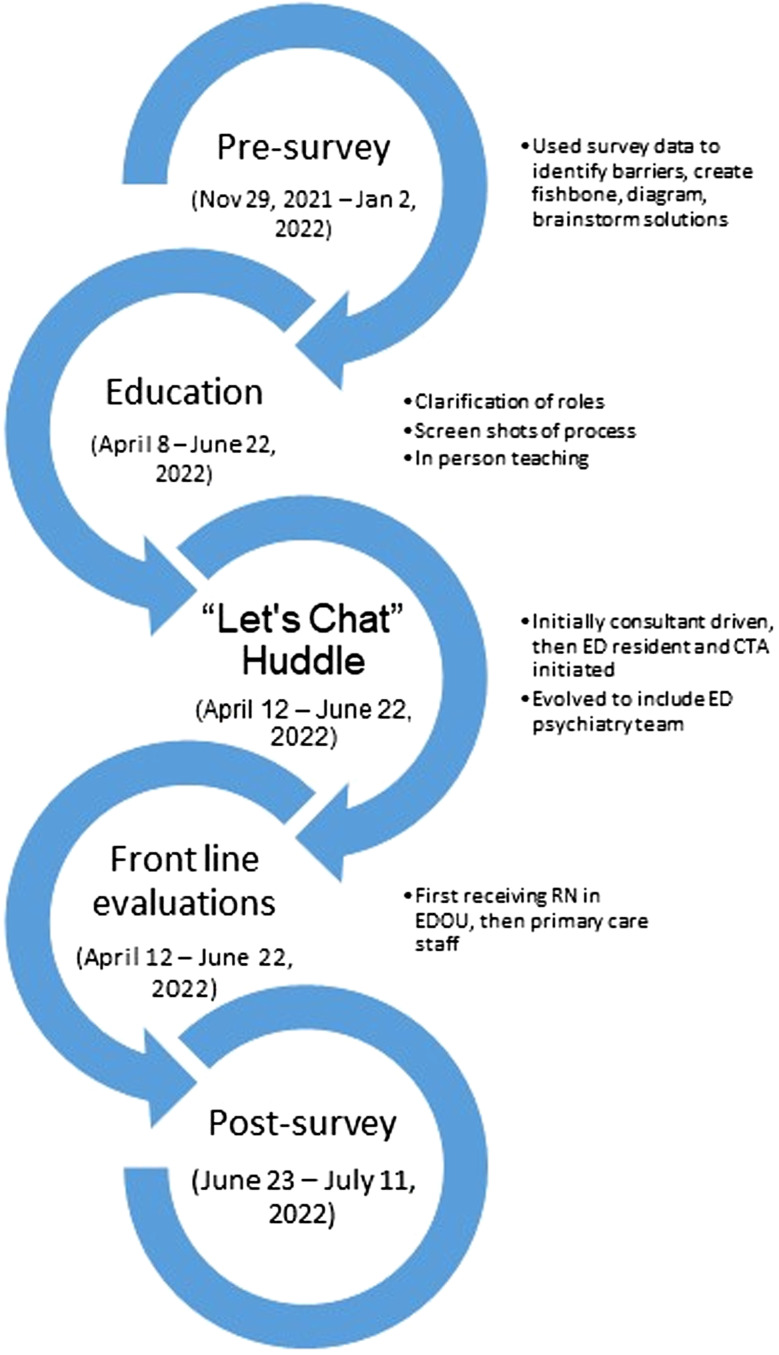
Plan, do, study, act (PDSA) cycles. *EDOU*, emergency department observation unit.

Figure 1 illustrates the timeline and summary of our project and the multiple plan-do-study-act (PDSA) cycles.

#### Pre-intervention survey

**Figure 2.**
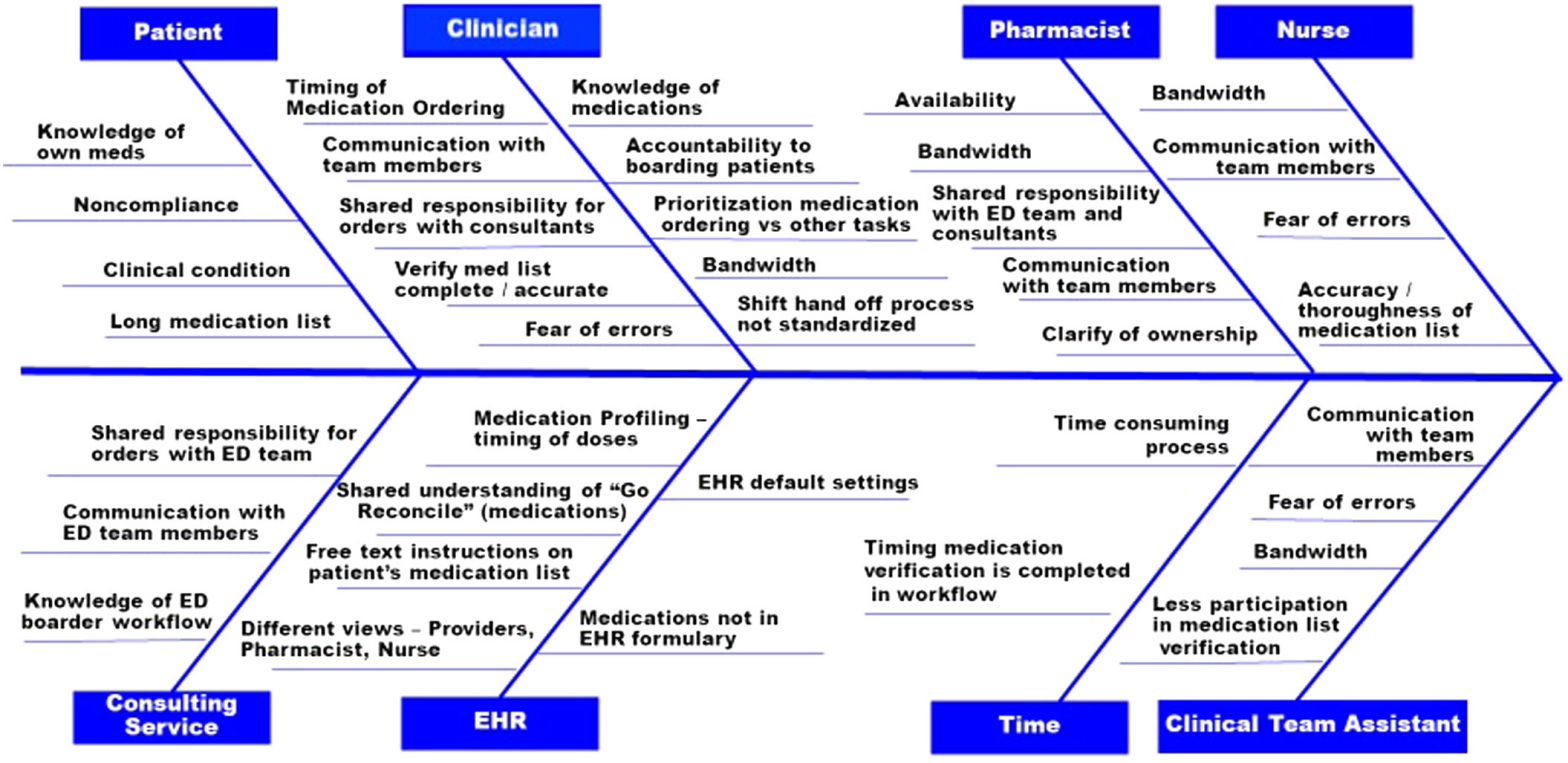
Fishbone diagram: stakeholder dissatisfaction with components of the medication reconciliation process for patients boarding in the emergency department. *ED*, emergency department; *EHR*, electronic health record.

In the first quarter of 2022, ED staff (emergency physicians and residents, NPs, PAs, pharmacists, and CTAs) received anonymous electronic surveys (Supplement 1). The survey was designed specifically to gauge satisfaction with initial medication history and medication reconciliation when the patient changes status to ED observation/BH boarding and to identify barriers to the process. Staff members in the ED rated satisfaction on a 4-point Likert scale (very dissatisfied = 0 to very satisfied = 3). From this survey, we identified potential gap(s) and their root causes, from the stakeholders’ viewpoints (Figure 2). We then focused on determining which key causes were amenable to improvement. Communication with care team members was identified as the underlying contributing factor that was most amenable to a process improvement.

The survey and its associated data were generated using Qualtrics software, version November 2021 (Qualtrics International Inc, Provo, UT).^17^

#### Electronic Health Record Alert

With knowledge gained from the baseline survey, the first proposed step to ameliorate the gap in care was an alert within the EHR to the patient care team. This pop-up would notify the associated ED team members to perform a medication reconciliation once the patient’s status was changed from “in process” to ED observation/BH boarding. This proposal was initially declined given limited availability of EHR programming resources during the pandemic.

#### Front-line Staff Education

**Figure 3.**
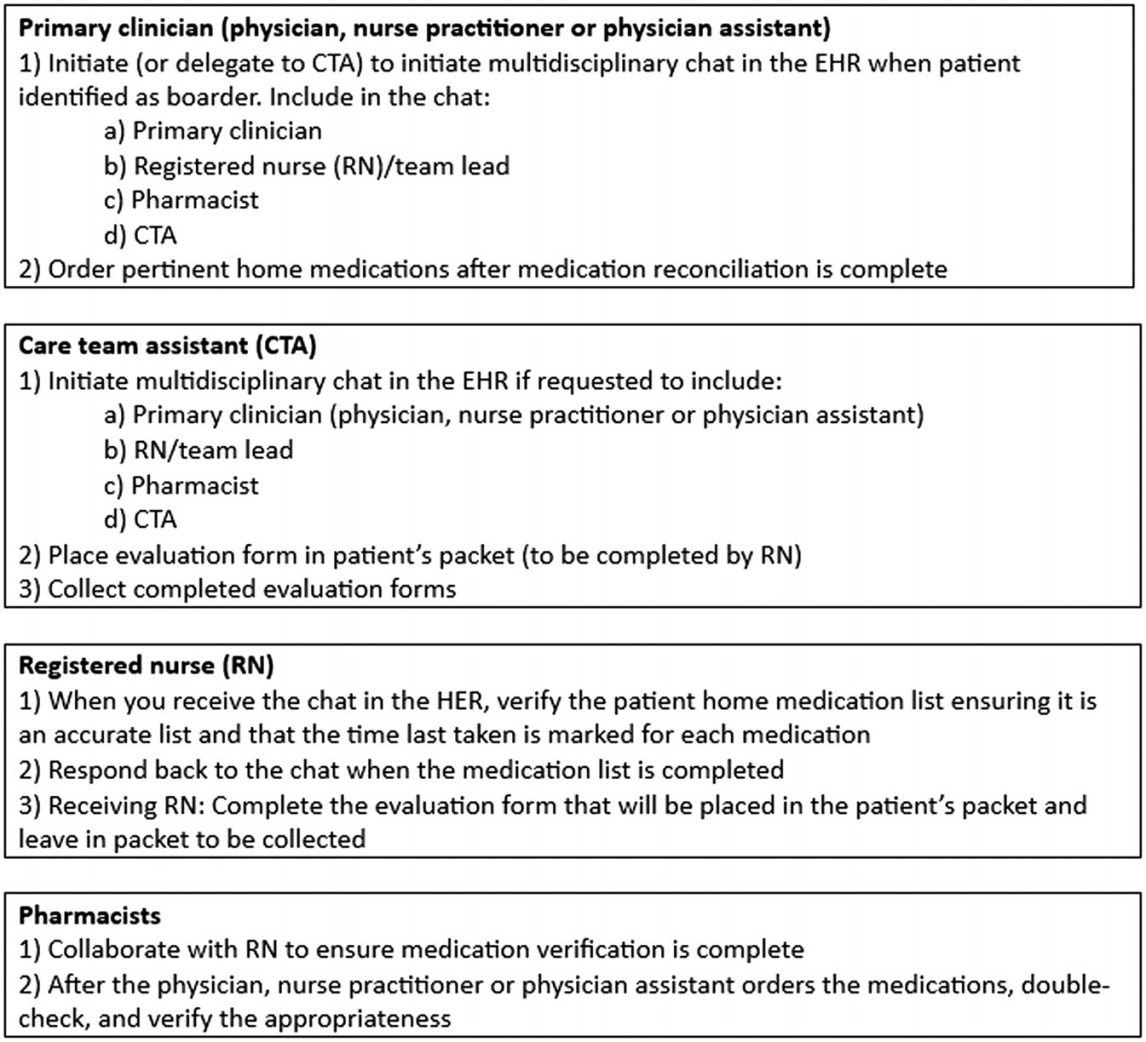
Educational document outlining role-specific tasks. *EHR*, electronic health record.

**Figure 4.**
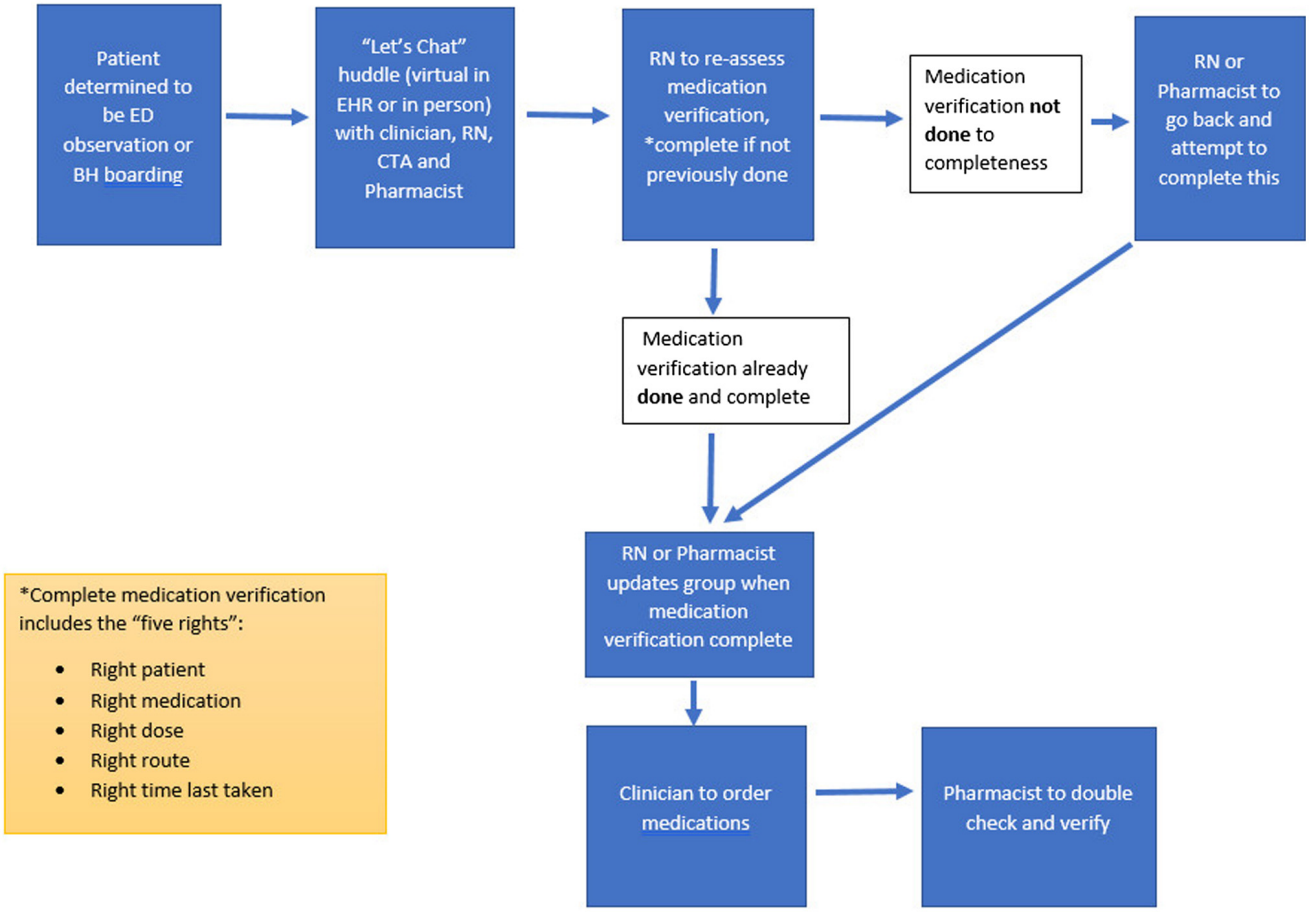
Flow diagram. *ED*, emergency department; *EHR*, electronic health record; *RN*, registered nurse; *CTA*, care team assistant.

In the pre-intervention survey, staff members noted a lack of clear delineation of roles for the medication history and reconciliation process. For the PDSA cycle starting on April 8, 2022, educational materials were created for staff members to delineate role-specific tasks (Figure 3) as well as identify a linear timeline of how the process of medication history and reconciliation should be completed to allow for time-efficient and safe patient flow in the ED (Figure 4). This new process included role-specific tasks for each ED team member that were optimized for their job-specific responsibilities and was designed so that medication orders for EDOU and BH boarding patients could be verified by a pharmacist and errors minimized.

The optimal flow was the CTA starts a “Let’s Chat” huddle, the bedside nurse completes the medication history, the primary clinician orders the medications based on the completed medication history, and the pharmacist then verifies the medication orders against the completed medication history. This medication reconciliation process was an additional responsibility given to ED team members who were already working; thus, our project did not require any additional hiring or full-time equivalennts. These materials were distributed to staff in the form of emails and handouts that were displayed throughout the ED for the duration of the initial intervention (April–July 2022). Education also included instructions on how to initiate a “virtual” multidisciplinary chat with active ED care team members—a “Let’s Chat!” huddle—within the EHR (Supplement 2). Staff also had the ability to have a huddle in person, if they preferred.

#### Attending physician-led “Let’s Chat!” Huddle

The ED attending physician is in charge of the patient’s care and has the most responsibility. Additionally, the attending physician is most familiar with medications and the plan of care for the patient’s ED course. The first intervention of the “Let’s Chat!” huddle required the attending physician to send the invitation to the care team. The investigators sent reminder emails, presented at the department meeting, and had in-person discussions with staff to encourage participation. This was met with resistance, as attendings were already taking on a large workload managing care for several patients while supervising and teaching. These factors led in many cases to the “Let’s Chat!” huddle not taking place and the medication reconciliation not being performed optimally. Regardless, our team felt that it was important to have the attending physician comfortable and familiar with this process as the team leader in the first iteration before we transitioned this responsibility to others.

#### Resident/NP/PA-initiated “Let’s Chat!” Huddle

In the next PDSA cycle, started on June 8, 2022, the resident physician, NP, or PA (whoever was caring for the patient), was tasked with initiating the “Let’s Chat!” huddle. These team members have similar knowledge of the patient’s medical history and treatment plans but oversee fewer patients at a time compared to the attending physician, which theoretically would allow the NP/PA/resident physician more time to initiate a multidisciplinary “Let’s Chat!” huddle Our department is a teaching institution and regularly has off-service residents rotating through the department. Often, these residents do not have the time to educate themselves on the medication history and reconciliation process during their brief time in the ED. For this reason, off-service residents were not expected to initiate the “Let’s Chat!” huddle; instead, ED residents and attending physicians helped them complete this process.

#### Care Team Assistant-initiated “Let’s Chat!” Huddle

In the final cycle (June 2022), CTAs initiated the “Let’s Chat” huddle. Our ED CTAs have overall awareness of the entire department and facilitate communication among team members, making them excellent at facilitating this process. With great response, CTAs were able to start the huddle promptly after noting the patient’s status change to ED observation or BH boarding in the EHR. This combined approach of a CTA-initiated electronic “Let’s Chat!” huddle to alert the nurse, clinician(s), and pharmacist to complete the medication history and reconciliation, and the subsequent roles each team member assumed allowed for designated multidisciplinary roles in the medication reconciliation process.

#### Emergency Department Psychiatry Consult Team Involvement

During our final cycle (June 2022), the ED psychiatry consult team also became involved in the “Let’s Chat” huddle for patients changing to BH boarding status. They were instructed to participate in the virtual or in-person huddle with the rest of the care team members. They were expected to weigh in on the psychiatric medications ordered for the patient. The ED psychiatry team showed enthusiastic participation in this process.

### Study of the Interventions

During each PDSA cycle, we used real-time evaluations by front-line ED staff (attending physicians, residents, NP/PAs, RNs, and pharmacists) to evaluate the effectiveness of each intervention cycle, obtain feedback on the process, and to determine how accurately medications were ordered (Supplement 3). This was initially done by the receiving nurse in the EDOU (whether BH boarding or ED observation patient) but was expanded to include all front-line ED staff. We used this information informally to adjust each PDSA cycle. This served a dual purpose as it was also a reminder to staff to do the “Let’s Chat!” huddle.

### Measures

We initially looked at hundreds of charts to identify quantitative indicators of errors or adjustments of medication reconciliation. Despite significant time dedicated to this data extraction, ultimately no useful quantitative data was obtained. Most of these errors are identified and corrected in real time through phone calls and in-person discussions, making it difficult to capture errors or adverse events using a retrospective health record review.

Our team reviewed the literature to see how others had obtained this data in similar projects, but there is a paucity of information regarding medication reconciliation in the ED. In studies of the medication reconciliation in inpatient units, review is frequently done by a pharmacist or pharmacy technician. Due to the limitations based on state law we were unable to use a pharmacy technician in the ED. Additionally, inpatient units lend themselves to better retrospective communication as the teams are more consistent day to day, allowing the pharmacist to ask the team about decisions made the day previously, whereas in the ED our teams are highly variable from shift to shift.

We also considered doing a quantitative review of reported medication errors or patient safety events during the time before and after our intervention. This was felt to be inaccurate as not every event gets reported. Due to our inability to identify a reliable quantitative measure of errors or safety events, we decided to focus on ED staff satisfaction. The thought was that if staff are satisfied and engaged in the process, there will be fewer errors. Front-line ED staff as stakeholders completed real-time evaluations to evaluate effectiveness during the PDSA cycles, provide feedback on the process, and completed the pre- and post-intervention surveys.

### Analysis

The same 4-point Likert scale was used for the post-intervention survey. Survey participants were asked their role in the ED, but this was de-identified from the rest of the responses for each survey. Responses were combined for analysis, no matter the role in the ED, to reflect the multidisciplinary nature of the impact of this study. We report averages of scores and overall satisfaction with the medication reconciliation process. Additionally, stakeholders were asked to provide free-text input about potential root causes of the gap in satisfaction. Each survey item was summarized with frequency counts and percentages for each response, as well as the overall mean response. We compared responses between the pre- and post-intervention surveys using two-sided Wilcoxon rank-sum tests and presented them as differences in proportions with 95% confidence intervals (CI). For each component of the medication history and reconciliation process, we used the average of the sum of “satisfied” or “very satisfied” responses to quantify the overall percentage staff satisfaction pre- and post-intervention.

## RESULTS

In April 2022, our team initiated the “Let’s Chat!” huddle to improve staff satisfaction with the medication history and reconciliation process. We administered a pre-intervention survey that was completed by 111 of 350 (31.7%) front-line ED staff across disciplines. (One staff member did not identify their role). In June 2022, we administered post-intervention surveys that were completed by 89 (25.4%) front-line staff. Completion rates are summarized in Table 1.

**Table 1.** Pre- and post-intervention survey completion rates of front-line staff.

Pre-intervention survey (number and percentage of front-line staff members responding)	Post-intervention survey (number and percentage of front-line staff members responding)
Physician (37/77 [48.1%])	Physician (39/77 [50.7%])
NP/PA (9/12 [75%])	NP/PA (8/12 [66%])
RN (54/150 [36%])	RN (33/150 [22%])
Pharmacist (10/10 [100%])	Pharmacist (9/10 [90%])

*Note:* Based on 110 respondents who identified their role.

*NP*, nurse practitioner; *PA*, physician assistant; *RN*, registered nurse.

### Pre-intervention Surveys

The pre-intervention survey identified a gap in ED staff satisfaction with the medication history and reconciliation process. In large part, staff were very dissatisfied with the medication reconciliation process for boarding patients. We looked specifically at each part of the “five rights” of medication administration: right patient; right medication; right dosage; right route; and right time.^18^ We found that 70.6% were dissatisfied or very dissatisfied with the right dosage, and 82.7% with the right time (time medication last taken).

### Post-intervention Surveys

After multiple interventions (see PDSA cycles above), the same survey was distributed to the same ED staff. Survey responses for each item are summarized in Table 2. Some respondents failed to answer each aspect of the survey, causing the individual totals of each question at times to add up to less than our total number of respondents. Respondents reported higher satisfaction with the medication reconciliation process after the intervention with regard to getting the right medication (1.69 vs 1.30; *P* = 0.004), right dosage (1.51 vs 1.03; *P* < 0.001), and time medication was last taken (1.29 vs 0.81; *P* < 0.001). Survey respondents were more satisfied with the medication history and reconciliation process getting the right patient prior to the intervention (average response 2.31 vs 2.16; *P* = 0.02), likely attributed to high satisfaction at baseline. There was no difference in satisfaction with the medication reconciliation process getting the right route for medication between the two surveys (*P* = 0.94).

**Table 2.** Summary of survey results.

	Very dissatisfied (0)	Dissatisfied (1)	Satisfied (2)	Very satisfied (3)	Average response	*P*-value
Right patient						
Pre-intervention	13 (11.7%)	9 (8.1%)	20 (18.0%)	69 (62.2%)	2.31	0.02
Post-intervention	5 (6.2%)	6 (7.4%)	41 (50.6%)	29 (35.8%)	2.16	
Right medication						
Pre-intervention	24 (21.8%)	40 (36.4%)	35 (31.8%)	11 (10.0%)	1.30	0.004
Post-intervention	8 (10.0%)	22 (27.5%)	37 (46.3%)	13 (16.3%)	1.69	
Right dosage						
Pre-intervention	35 (32.1%)	42 (38.5%)	26 (23.9%)	6 (5.5%)	1.03	<0.001
Post-intervention	9 (11.3%)	27 (33.8%)	38 (47.5%)	6 (7.5%)	1.51	
Right route						
Pre-intervention	21 (19.1%)	17 (15.5%)	36 (32.7%)	36 (32.7%)	1.79	0.94
Post-intervention	6 (7.5%)	12 (15.0%)	47 (58.8%)	15 (18.8%)	1.89	
Time medication was last taken						
Pre-intervention	45 (40.9%)	46 (41.8%)	14 (12.7%)	5 (4.5%)	0.81	<0.001
Post-intervention	13 (16.5%)	33 (41.8%)	30 (38.0%)	3 (3.8%)	1.29	

*Note:* Based on 111 responses received for the pre-intervention survey and 89 responses received for the post-intervention survey.

When we combined the percentage of respondents choosing “satisfied” or “very satisfied” and compared pre- to post-intervention satisfaction with the medication history and reconciliation process, we also saw an overall improvement in satisfaction (as shown in Table 3). Three of the “five rights” of the components of medication reconciliation had improvement in staff satisfaction over our stated goal of 20%. Overall, we saw a 17.9% improvement in ED staff satisfaction (64.7% vs 46.8%).

**Table 3.** Staff satisfaction with each component of the “5 rights”.

Survey question	Pre (percentage responding satisfied or very satisfied)	Post (percentage responding satisfied or very satisfied)	Change in percentage meeting satisfaction criteria	>20% threshold met
Satisfaction with medication reconciliation when the patient’s status changes to ED observation/BH boarding	Right patient (80.2%)	Right patient (86.4%)	6.2%	No
	Right medication (41.8%)	Right medication (62.6%)	20.7%	Yes
	Right dose (29.4%)	Right dose (55%)	25.6%	Yes
	Right route (65.4%)	Right route (77.5%)	12.1%	No
	Time last taken (17.3%)	Time last taken (41.8%)	24.5%	Yes
Overall percent satisfaction	46.8%	64.7%	17.9%	No

In free-text responses in the post-intervention survey, many staff members noted that increased use of the “Let’s Chat!” huddle was felt to be an additional venue through which all team members, knowing their roles in the process, can assist one another to ensure that medication reconciliation is complete and accurate.

## DISCUSSION

### Summary

Patients are experiencing increasing LOS in the ED.^2^ During these prolonged stays, patients require medication history reconciliation^1^; unfortunately this process is complicated and challenging, leading to ADE.^8^ Delineation of roles and the electronic chat function in the EHR (“Let’s Chat!” huddle) were novel interventions that led to measurably increased satisfaction with the medication history and reconciliation process for EDOU and BH boarding patients. Using validated frameworks like the Lean Six Sigma, this project increased the understanding of how to improve the quality of ED care for BH boarding and EDOU patients.^19^

A chat function within the EHR allowed for alternative means of communication and increased the flexibility and buy-in of ED staff members. Evident in the low return of responses to the post-intervention surveys, there is a steep learning curve to get a large number of multidisciplinary staff educated on this new process in a busy work environment to implement the change.

### Interpretation

Looking at this system as a whole, the “Let’s Chat!” huddle improved front-line staff satisfaction with the medication reconciliation process, which should correlate with improved patient safety, decreased LOS, and positive patient outcomes.^20^ Measuring satisfaction in specific aspects of this process taps into the multidisciplinary nature of medication history and reconciliation and covers many bases that could be missed with a solitary unit of measurement (eg, LOS, ADEs). Measurement of staff satisfaction allows the stakeholders to apply their judgment as to whether the process was a success or failure, serving as a “stamp of approval” with the process.

This novel study is difficult to compare to other research, given the lack of published QI work covering this topic. Availability of pharmacy technicians is a focal point of prior studies; however, due to state statutes we were unable to use this group in our ED.^9^ In attempting to facilitate a change, the efforts of the “Let’s Chat!” huddle found that a collaborative multidisciplinary approach is necessary to have impact in this process. Carpenter et al demonstrated that knowledge alone is necessary but insufficient to improve healthcare outcomes; thus, adapting behaviors of clinicians, patients, and stakeholders to new standards of evidence-based clinical practice is often significantly delayed.^21^

Future directions for research include working on an implementation study with evidence-based interventions, determining how to measure patient-oriented health outcomes, testing the effectiveness of the implementation strategy, and including cost analysis, fidelity of the intervention, and evaluation of unintended effects in groups, among other steps as recommended by the Standards for Reporting Implementation Studies statement.^22^

The “Let’s Chat!” medication reconciliation process was approved as a practice at this institution going forward. After the proven success of the project, the EHR alert has been implemented, alerting CTAs to initiate a “Let’s Chat!” huddle when patients are placed on boarding status. This automated process could potentially be applied for discharging patients as well, which would broaden its impact and further decrease ED LOS.

The engagement and awareness of the team involved in the medication history and reconciliation process is critical to the safety of our patients, staff satisfaction, and optimal outcomes. Attention to the medication history and reconciliation continues to be an important part of the patient’s ED visit. Continued reinforcement of the interventions, communication with staff, and monitoring for safety events is needed in the future to determine whether actual improvement is recognized by staff.

## LIMITATIONS

Because this was a single-center study it may not be inherently generalizable to other institutions with fewer ED staff resources. Second, staff satisfaction is impacted by many factors that are not possible to measure or control. There were low response rates (from 25.4–31.4%) with the lowest completion rate among nurses who are our largest and most heterogeneous group of ED staff. We should also acknowledge that staff in the email list were not all working clinically during the four-week period that the survey was open.

Third, the sampling population was limited, as the survey was elective. This may have contributed to participation bias from individuals with strongly weighted feelings toward this process to skew the results. Additionally, overall satisfaction with this process is difficult to conclude, as an improved ED medication reconciliation extends beyond the front-line ED staff to the inpatient and consulting psychiatry teams, hospitalists, and patients who were not surveyed for their satisfaction and potential feedback. A wider net could be cast in the future iterations of this project to avoid survivorship bias.

Fourth, by using staff satisfaction instead of measurable quantitative information about errors or safety events related to medications reconciliation, the data is subject to the responders’ interpretation of the question. Quantitative data is difficult to sway in this fashion and is a limitation of using satisfaction. Fifth, resistance and intermittent failure of ED staff to perform “Let’s Chat” huddles during the physician-led huddle cycle due to lack of familiarity with roles could mean that the two-month window for staff to be familiarized with the intervention may have been insufficient for them to comfortably use the new process before answering the post-intervention survey. Historically, other implementation strategies have demonstrated an initial enthusiasm by staff that swiftly wanes. Use of a washout period between interventions could prevent this attrition and allow for more time for staff to passively review information while not having to use it. Further experience and use of the “Let’s Chat!” huddles, if sustained, will allow staff to become more comfortable with the process.

Sixth, the method of staff education (email and printed materials) was selected based on availability of resources and not the most effective method backed by research for distributing information and educating a team. Further work should include evaluation of the sustainability of the “Let’s Chat!” virtual huddle tool, duration of the effectiveness of education strategies used, and application to other patient groups dismissed from the ED.

## CONCLUSION

The “Let’s Chat!” huddle facilitates communication and increases satisfaction among ED team members related to the medication reconciliation process. The increased use of the “Let’s Chat!” huddle was felt to be an additional and effective venue through which all team members, knowing their roles in the process, can assist one another to ensure the medication reconciliation is complete and accurate. Ongoing work is needed to continue to improve and build on the culture change for enhancing the medication history and reconciliation process.

## Supplementary Information




