# Supplementary material for: “Let’s Chat!” Improving Emergency Department Staff Satisfaction with the Medication Reconciliation Process
Source: West J Emerg Med. 2024 May 21;25(4):624–33. doi: 10.5811/westjem.18324 (PMC11254147; doi:10.5811/westjem.18324)
Supplement: Supplementary file 1 [file wjem-25-624-s001.pdf]

# Supplemental Materials Item 1: Pre/Post-Intervention Survey

This is the survey distributed to Emergency Department employees filled out to evaluate satisfaction with the "Let's Chat!" Project.

# Med Rec for Long-Stay Patients: Baseline Survey

Start of Block: Default Question Block

Q1 What is your role in the ED?

○ Physician (1)

○ NP/PA (2)

○ RN (3)

○ Pharmacist (4)

Q2 Rate your satisfaction with the current medication reconciliation process-Initial verification/clarification of patient medication list.

| | 0=Very Dissatisfied (1) | 1=Dissatisfied (2) | 2=Satisfied (3) | 3=Very Satisfied (4) |
|---|---|---|---|---|
| Right patient (1) | ○ | ○ | ○ | ○ |
| Right medication (2) | ○ | ○ | ○ | ○ |
| Right dose (3) | ○ | ○ | ○ | ○ |
| Right route (4) | ○ | ○ | ○ | ○ |
| Time last taken (5) | ○ | ○ | ○ | ○ |

Q3 Rate your satisfaction with the current medication reconciliation process- When the patient changes to EDOU or boarded status.

| | 0=Very Dissatisfied (1) | 1=Dissatisfied (2) | 2=Satsified (3) | 3=Very Satisfied (4) |
|---|---|---|---|---|
| Right patient (1) | ○ | ○ | ○ | ○ |
| Right medication (2) | ○ | ○ | ○ | ○ |
| Right dose (3) | ○ | ○ | ○ | ○ |
| Right route (4) | ○ | ○ | ○ | ○ |
| Timing of ordered med(s) to be given (5) | ○ | ○ | ○ | ○ |

Q4 What are the barriers to the medication reconciliation process for EDOU/Boarded Behavioral Health patients?

| | 0=Not a barrier (1) | 1=Barrier less than 50% of the time (2) | 3=Barrier more than 50% of the time (3) | 4=Significant barrier (4) |
|---|---|---|---|---|
| (Time) Amount of time it takes to complete a thorough medication history. (1) | ○ | ○ | ○ | ○ |
| Discrepancy of practice between the ED versus inpatient setting (2) | ○ | ○ | ○ | ○ |
| Patients and/or family do not know their | ○ | ○ | ○ | ○ |

| | | | | |
|---|---|---|---|---|
| medication history (3) | | | | |
| Knowledge around the medication reconciliation process (4) | ○ | ○ | ○ | ○ |
| Understanding of "Go Reconcile" feature (5) | ○ | ○ | ○ | ○ |
| Timing of EDOU/Boarding patient medication ordering (6) | ○ | ○ | ○ | ○ |

Q5 What are the barriers to the medication reconciliation process for EDOU/Boarded Behavioral Health patients with incomplete medication history.

| | 0=Not a barrier (1) | 1=Barrier less than 50% of the time (2) | 3=Barrier more than 50% of the time (3) | 4=Significant barrier (4) |
|---|---|---|---|---|
| Missing/Incorrect medication (1) | ○ | ○ | ○ | ○ |
| Missing/Incorrect dose (2) | ○ | ○ | ○ | ○ |
| Missing/Incorrect route (3) | ○ | ○ | ○ | ○ |
| Missing/Incorrect time last taken (4) | ○ | ○ | ○ | ○ |

Q6 Rate the degree to which the following communication issues add to confusion or barriers to an accurate process.

| | 0=Not a barrier (1) | 1=Barrier less than 50% of the time (2) | 3=Barrier more than 50% of the time (3) | 4=Significant barrier (4) |
|---|---|---|---|---|
| Communication between all care team members about the plan (1) | ○ | ○ | ○ | ○ |
| Shared responsibility of order writing between ED and Psychiatric team (2) | ○ | ○ | ○ | ○ |
| Shared responsibility of order writing between ED and Pharmacy team (3) | ○ | ○ | ○ | ○ |
| Ensuring the medication list is complete before ordering (4) | ○ | ○ | ○ | ○ |

Q7 Other/More details regarding answers from above.

________________________________________________________________

________________________________________________________________

________________________________________________________________

Q8 How can the medication reconciliation process for EDOU/Boarded Behavioral Health patients be improved?

________________________________________________________________

________________________________________________________________

________________________________________________________________

# Supplemental Materials Item 2:
# How to Start a Chat in Epic:

This was distributed and made available to Emergency Department employees during the entirety of the Let's Chat! Project.

1. On the track board, **hover** over any of the **care team members** (Attending, NP/PA/Res, RN) and **double click**

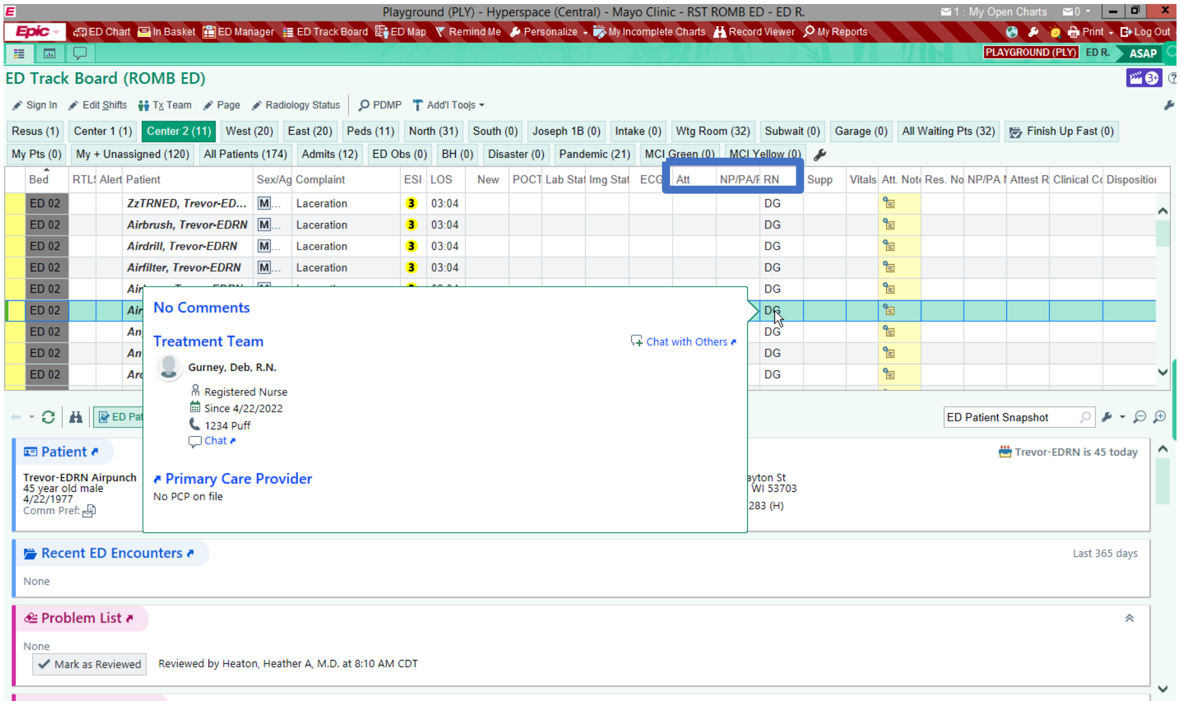


2. A screen will pop up where you can select " **chat with all active members** "

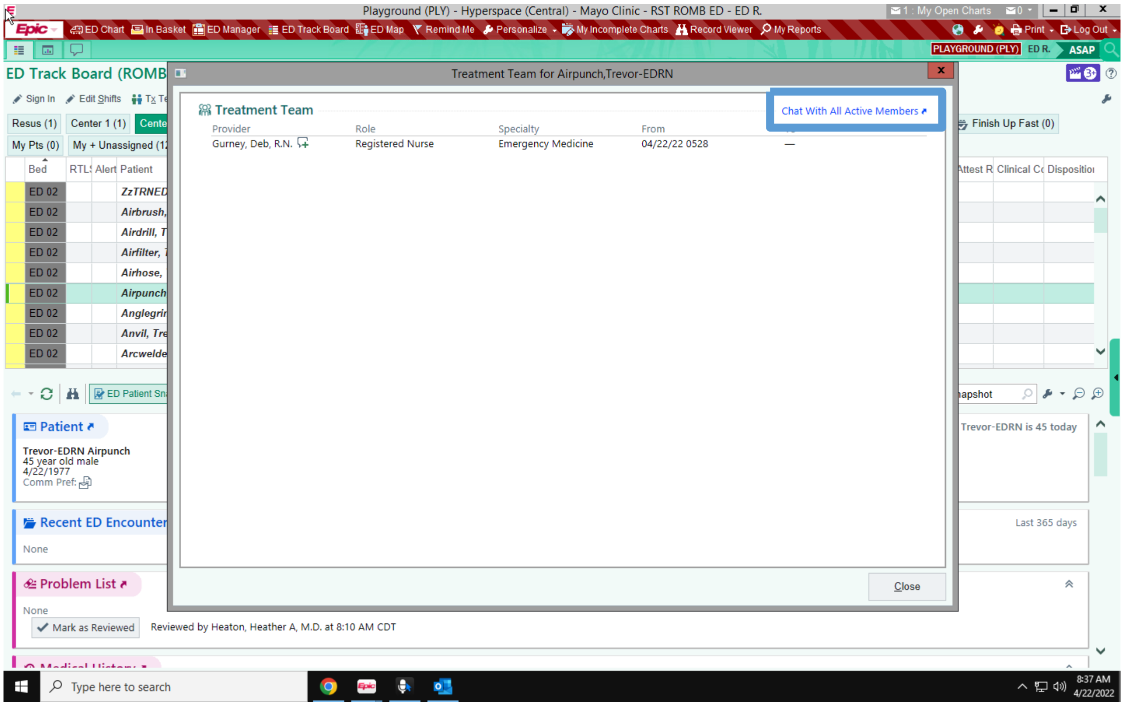

3. Start the chat. Current care team members (RN, consultant, resident, NP/PA) will already be included.

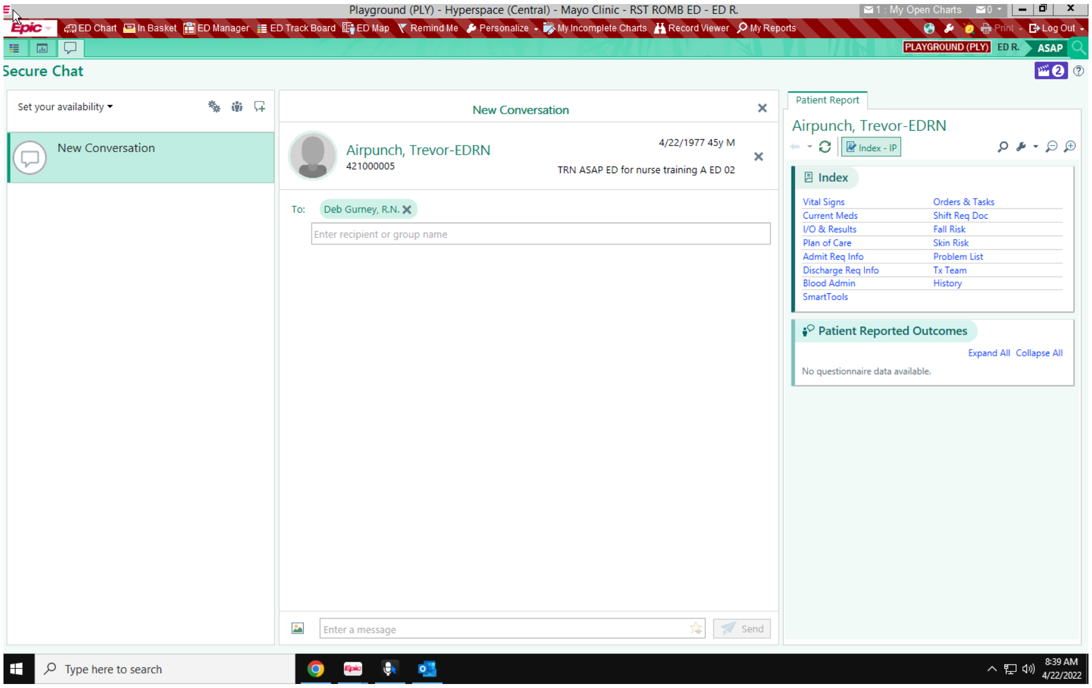


4. Add in Pharmacy – can add them **individually by name** or via the **group**. Search "RST ED" and click on "**groups**"

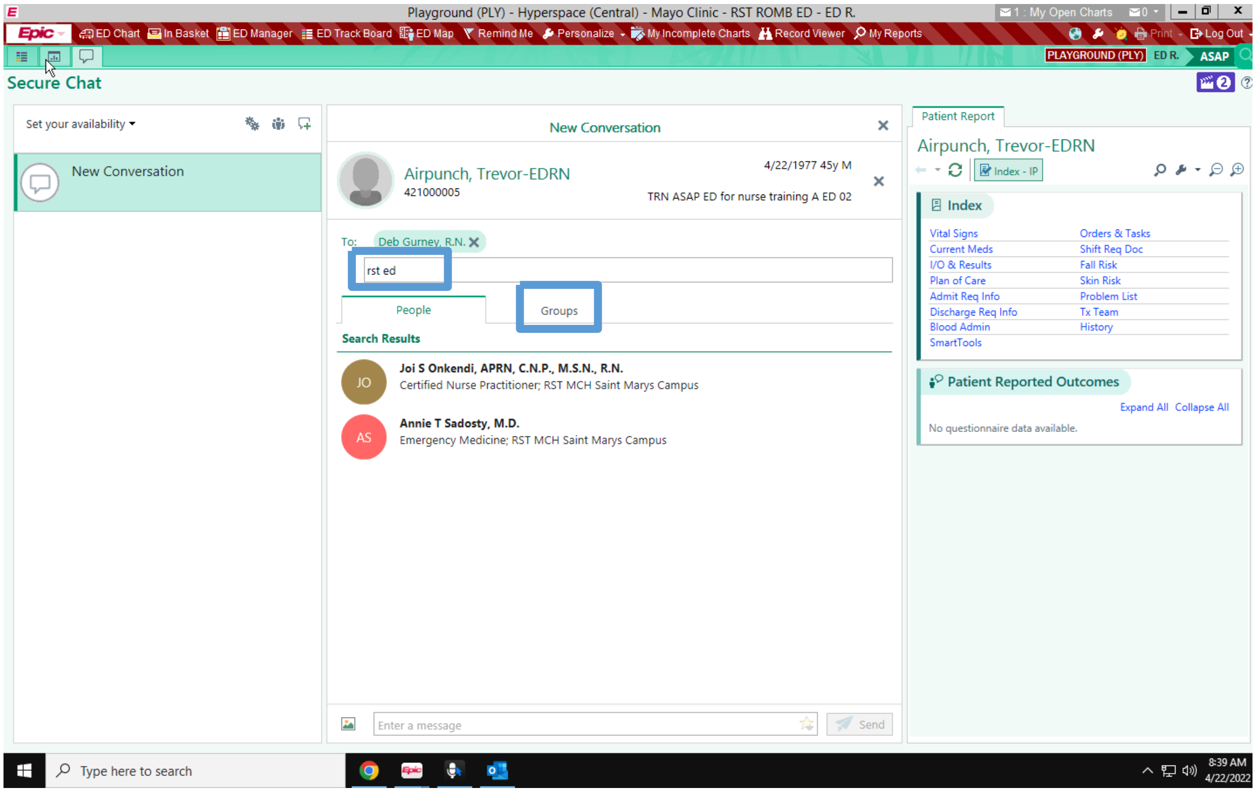

5. You can "**star**" the pharmacy group so it is easy to find next time

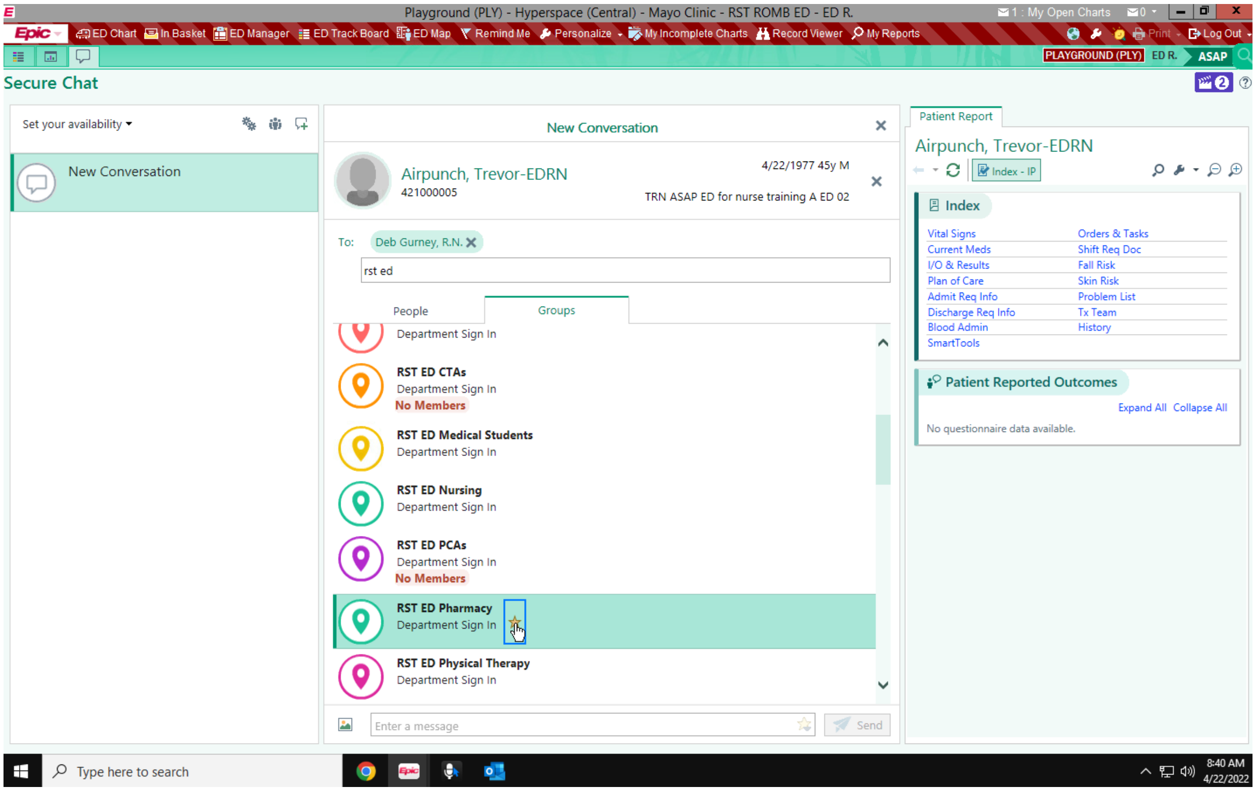


6. All done!

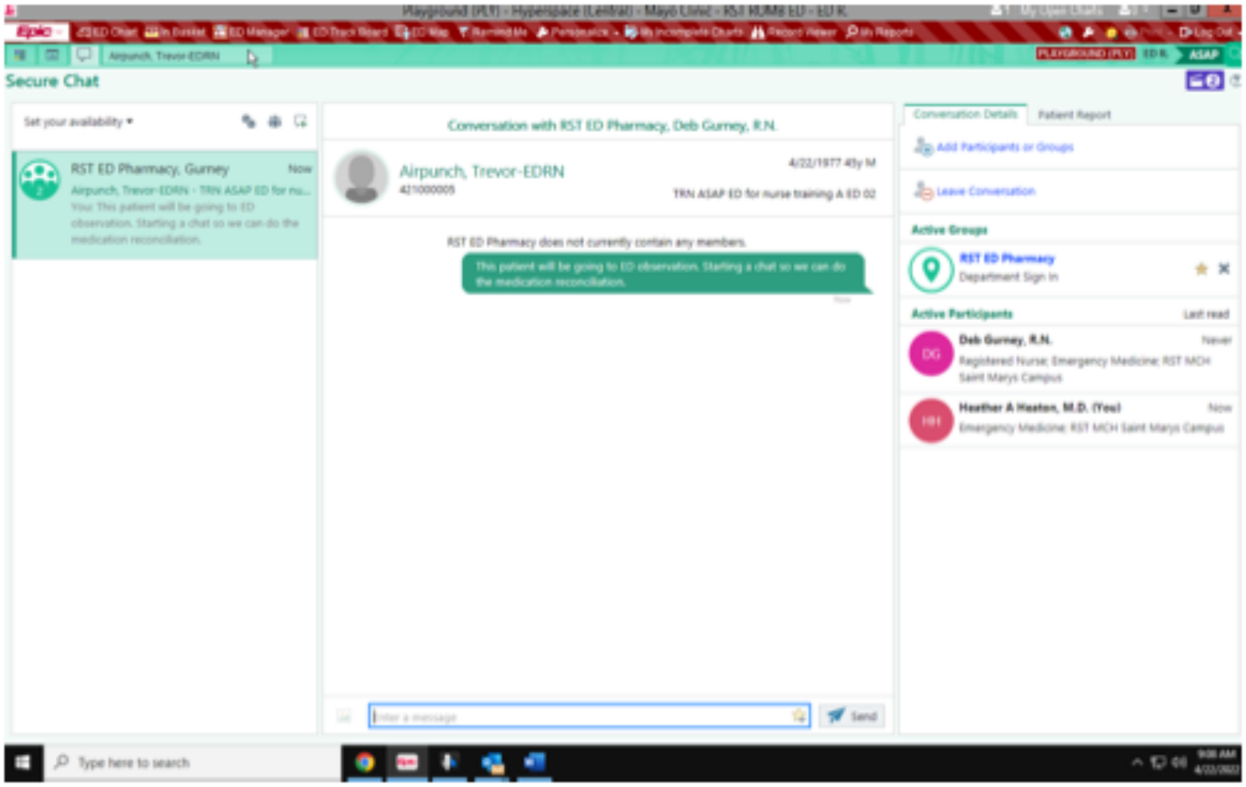

# Supplemental Materials Item 3:
# Pilot Intervention Evaluation Form:

This was distributed and made available to Emergency Department employees during the entirety of the Let's Chat! Project.

Place patient sticker here

## EDOU/BH Boarder Medication Reconciliation Pilot Intervention Evaluation

1. Rate your satisfaction with how accurately the patient's home medications were ordered

| 1 | 2 | 3 | 4 |
|---|---|---|---|
| Not satisfied | Somewhat satisfied | Satisfied | Very satisfied |

2. Did you need to correct any medication orders? Yes No
   a. If yes, how many medications needed correction?
   b. Please indicate what was incorrect with the medication order(s):

| Medication name | Missing medication | Incorrect medication | Incorrect dose | Incorrect route | Incorrect timing | Comments |
|---|---|---|---|---|---|---|
| | | | | | | |
| | | | | | | |
| | | | | | | |
| | | | | | | |

(continued on the back, if needed)

3. Rate the degree to which the following added confusion or barriers to accurate med ordering:

| | **Significant barrier** | **Barrier** | **Somewhat of a barrier** | **Not a barrier** |
|---|---|---|---|---|
| Communication between care team members | | | | |
| Shared responsibility of order writing | | | | |
| Ensuring the med list is complete before ordering | | | | |

**Return completed evaluation to patient's packet to be collected in Control**

Place patient sticker here

| Medication name | Missing medication | Incorrect medication | Incorrect dose | Incorrect route | Incorrect timing | Comments |
|---|---|---|---|---|---|---|
| | | | | | | |
| | | | | | | |
| | | | | | | |
| | | | | | | |
| | | | | | | |
| | | | | | | |
| | | | | | | |
| | | | | | | |
| | | | | | | |
| | | | | | | |
| | | | | | | |
| | | | | | | |
| | | | | | | |
| | | | | | | |
| | | | | | | |
| | | | | | | |

**Return completed evaluation to patient's packet to be collected in Control**
